# Impact of Vitamin D Injection on Keloids and Hypertrophic Scars

**DOI:** 10.1111/jocd.70118

**Published:** 2025-03-26

**Authors:** Amel R. Hassan, Ammena Y. Binsaleh, Samar M. El‐Tahlawi, Azza M. El‐Amir, Mary M. Ishak, Nawal Alsubaie, Thanaa A. El‐Masry, Mostafa M. Bahaa, Mamdouh Eldesoqui, Marwa Kamal

**Affiliations:** ^1^ Department of Dermatology, STDs and Andrology, Faculty of Medicine Fayoum University Fayoum Egypt; ^2^ Department of Pharmacy Practice, College of Pharmacy Princess Nourah bint Abdulrahman University Riyadh Saudi Arabia; ^3^ Department of Pharmacology and Toxicology, Faculty of Pharmacy Tanta University Tanta Egypt; ^4^ Pharmacology and Toxicology Department Faculty of Pharmacy, Sinai University, Arish campus Egypt; ^5^ Department of Pharmacy Practice Faculty of Pharmacy, Horus University New Damietta Egypt; ^6^ Department of Basic Medical Sciences College of Medicine, AlMaarefa University Riyadh Saudi Arabia; ^7^ Department of Clinical Pharmacy, Faculty of Pharmacy Fayoum University Fayoum Egypt

**Keywords:** hypertrophic scars, keloids, vitamin D

## Abstract

**Background:**

Hypertrophic scars and keloids are human cutaneous fibroproliferative conditions that develop after burns, trauma, surgery, and inflammation. Vitamin D inhibits keloid fibroblast proliferation by reducing TGF‐β‐induced extracellular matrix formation, boosting matrix metalloproteinase activity, and reducing inflammation.

**Aim:**

To study the effect of intralesional and systemic Vitamin D3 injection on hypertrophic scars and keloids and whether vitamin D3 deficiency increases scarring.

**Patients and Methods:**

This study included 30 hypertrophic scars and keloid patients divided into groups depending on serum vitamin D levels. Every patient was tested for vitamin D using ELISA. Group I: patients with vitamin D deficiency or insufficiency received a systemic injection of vitamin D (cholecalciferol 200 000 I.U.) once monthly for 3 months with a calcium oral supplement and intralesional vitamin D injections on hypertrophic scars and keloids. Group II: patients with sufficient vitamin D received only intralesional vitamin D injections.

**Results:**

Vitamin D deficiency did not affect scar formation or severity (total Vancouver scar scale before assessment) with a *p* value > 0.05. All instances showed a substantial drop in vascularity, pliability, and total Vancouver scale score (*p* value < 0.05) following intervention, but no change in scar pigmentation or height. Scar assessment following intervention did not significantly differ between research groups (*p* > 0.05).

**Conclusion:**

Injection of vitamin on hypertrophic scars and keloids enhances vascularity and pliability in patients with sufficient serum vitamin D levels and those with deficient or insufficient serum vitamin D levels after improving them by systemic injection of vitamin D without any effect on height and pigmentation of scars.

**Trial Registration:**

NCT06301178

## 1 Introduction

A keloid is an aberrant scar that extends from the initial site of the skin lesion. Keloids typically cause discomfort and pruritus and present clinically as an elevated, amorphous growth [1]. Patients with scars and keloids experience major physical and psychological suffering. Keloids enlarge, whereas hypertrophic scars rise above the skin but remain within the scar borders [2].

Inadequate wound repair leads to the formation of keloids and hypertrophic scarring. Increased accumulation of extracellular matrix (ECM) distinguishes these lesions apart. Reduced remodeling processes and an increase in proliferative and inflammatory mechanisms lead to extensive ECM formation. Moreover, hereditary and systemic factors are connected to these scarring lesions [3].

Scar therapy for keloids is a difficult and contentious treatment. Due to their increased effectiveness over individual therapy methods, combined treatments are being used increasingly frequently [4]. Various therapies have been used; however, no scar therapy technique works. A variety of treatments are available, including the use of pressure garments, cryotherapy, lasers, radiation, and surgery, as well as bleomycin, interferon, 5‐fluorouracil (5‐FU), and the topical and intralesional injections of corticosteroids. Keloids and hypertrophic scars recur 50%–80% and 10%, respectively, despite various therapies [5].

In addition to modulating inflammatory processes, vitamin D also plays important regulatory functions in cellular growth and differentiation, wound repair, and other processes. The primary mechanism underlying these results is the interaction between activated vitamin D and vitamin D receptors (VDR), thereby turning on the control of gene transcription. Vitamin D has the ability to inhibit the growth of fibrous tissue, destroy fibroblast cells, and reduce the production of collagen [6].

Vitamin D has been shown to stop the growth of keloid fibroblasts by stopping the production of extracellular matrix triggered by TGF‐β and increasing the production activity of matrix metalloproteinase and working as an anti‐inflammatory mediator, so the study's goal was to find out if intralesional and systemic vitamin D3 injections can help treat hypertrophic scars and keloids, and to find out if not getting enough vitamin D3 is linked to a higher risk of scarring.

## 2 Materials and Methods

### 2.1 Study Design

This study was a clinical trial carried out on 30 individuals from the attendants of the Outpatient Clinic of the Dermatology, STDs, and Andrology Department. The study lasted from February 15, 2024 until July 20, 2024.

### 2.2 Study Population

**FIGURE 1:**
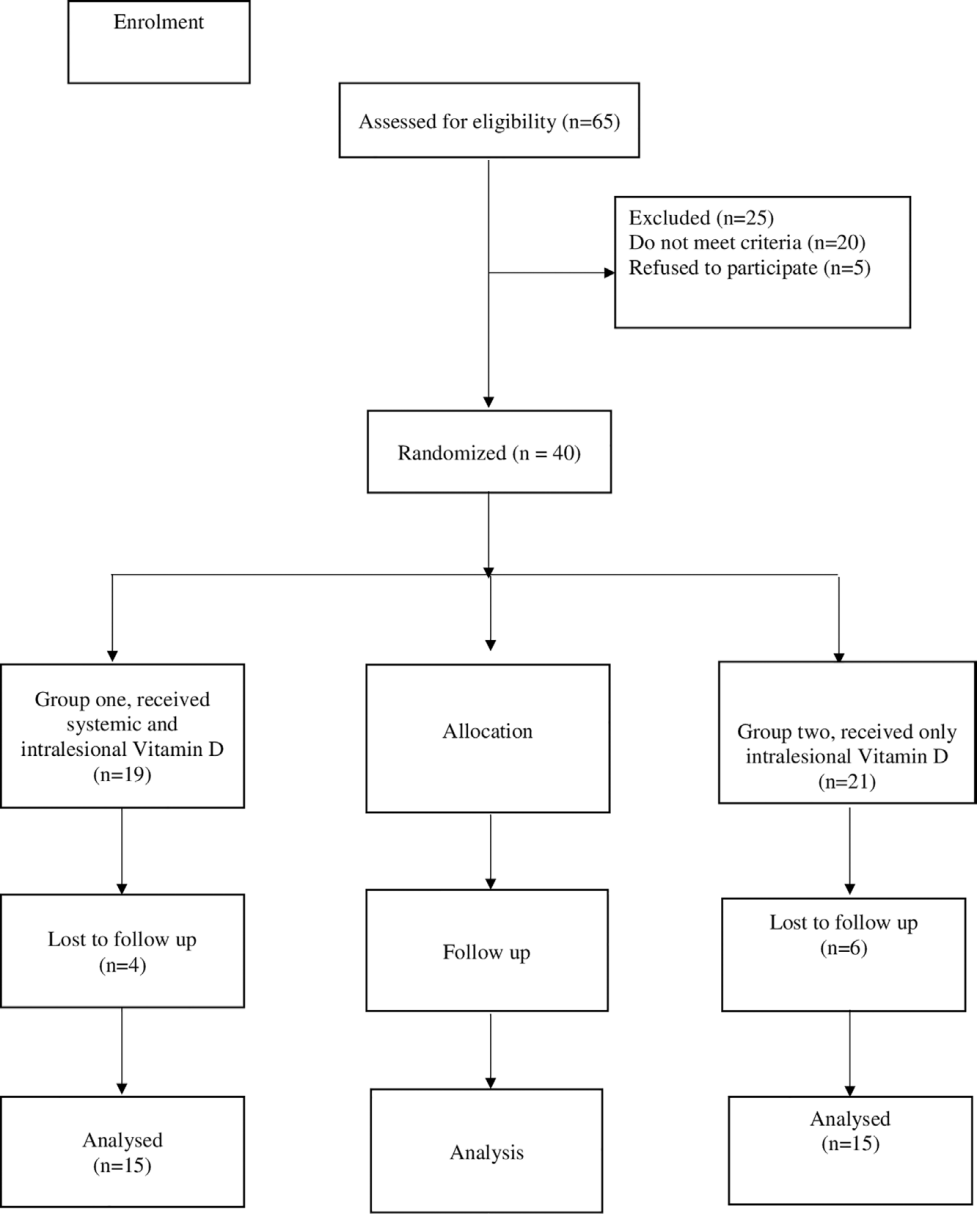
CONSORT diagram showing the disposition of participants during the study.

In the study, 30 patients with keloids and hypertrophic scars were included according to the CONSORT diagram (Figure 1). The diagnosis was based on clinical evidence.

### 2.3 Technique

#### 2.3.1 History Taking

– Cause and duration of the scar.
– Past history.
– Associated diseases.
– Associated itching.
– Previous treatment.

#### 2.3.2 General Examination

To exclude any systemic diseases that may affect the level of vitamin D.

#### 2.3.3 Dermatological Examination

– Site, extent, and nature of the scar.
– Scar condition, itching, fibrosis, calcification, or inflammation.

### 2.4 Inclusion Criteria

1. Age range: 12–50 years old.
2. Patients who had keloids and hypertrophic scars.

### 2.5 Exclusion Criteria

1. Below the age of twelve and above fifty
2. Patients underwent further modalities of therapy for keloids and hypertrophic scars.
3. Skin disorders, including systemic ones
4. The patients were already taking extra vitamin D supplements

### 2.6 Ethical Consideration

All participants had the right to participate in the study or not. All collected data were kept confidential. All patients were asked to give their verbal and written consent.

### 2.7 The Material Used

Vitamin D ampoules (Devarol‐S 200 000 I.U., Chemipharm, Mamphis, Egypt). Each pack contains a single 2 mL ampoule of these locally made products.

#### 2.7.1 Serum Sample Collection

Each patient in the study had 3 mL of blood drawn via venipuncture from the vein just below the ankle. The serum separation process began by collecting blood samples in plain vacutainer tubes.

The serum was separated by incubating the samples at 37°C for 10–15 min and then centrifuging at 3000 rpm.

#### 2.7.2 Determination of Vitamin D

As instructed by the manufacturer, an ELISA kit (Sigma‐Aldrich, St. Louis, MO, USA) is used to assay vitamin D.

#### 2.7.3 Vitamin D Injection

According to the level of vitamin D, patients were allocated and split into two groups:

Group I: Patients with deficiency or insufficiency of vitamin D (< 12 ng/mL and 12–19 ng/mL respectively) were given both systemic injections of vitamin D (Cholecalciferol 200 000 I.U.) once monthly for 3 months, along with a calcium oral supplement + intralesional injection of vitamin D on hypertrophic scars and keloids.

Group II: Patients with sufficiency of vitamin D (20 and greater ng/ml) had taken only intralesional vitamin D injection on hypertrophic scars and keloids.

#### 2.7.4 Intralesional Injection of Vitamin D

Using an alcohol pad to clean the injection site, an intralesional injection of non‐diluted cholecalciferol 200 000 I.U. using a 1‐mL U‐100 insulin syringe with a dose of 0.3 mL was performed, and to alleviate any discomfort, the injection site was gently compressed. There were four sessions, and they were held every 2 weeks after that. We chose areas in the scar that appear clinically affected measuring 3 cm × 3 cm, and we inject about 0.1 mL in each cm.

### 2.8 Methods of Evaluation

Clinical scoring by Vancouver Scale Score (VSS) (vascularity, pliability, pigmentation, and scar height or thickness).

### 2.9 Statistical Analysis

In order to manipulate the data, it was obtained, coded, and double‐entered into Microsoft Access. To analyze the data, Windows 10's Statistical Package for the Social Sciences (SPSS) 28 was utilized. Basic descriptive analysis measured quantitative parametric data dispersion with standard deviations, central tendency determined by arithmetic means, and qualitative data as percentages and numbers.

– Quantitative parametric data: To compare quantitative measurements between two independent groups, the unpaired *t*‐test was employed. The paired *t*‐test was used to compare two dependent quantitative data sets.
– For qualitative data: Use a chi‐square test to compare two or more qualitative groups.

To ascertain variable connections, use the Pearson bivariate correlation test.

Statistical significance was indicated by a *p* value less than 0.05.

## 3 Results

Thirty patients with hypertrophic scars and keloids were included. The duration of the scar in the study group I ranged between (2 months and 23 years) with a median and interquartile range of [5 (2–7)] and in the study group II ranged from (3 months to 15 years) with a median and interquartile range of [2 (0.75–10)]. There was no statistically significant difference between the two study groups, *p* = 0.571. Patients were divided into two groups, 15 in each group, according to vitamin D level.

### 3.1 Age Among Different Study Groups

Group I: Age ranged between 12 and 39 years with a mean 20.5 ± 8.4.

Group II: Age ranged between 12 and 33 years with mean 21.9 ± 7.2.

No statistically significant differences were observed (*p* = 0.6) regarding age between study groups as demographic characters (Table 1).

**TABLE 1:** Study group comparisons based on demographic variables.

Variables	Group I (*N* = 15)		Group II (*N* = 15)		*p*	Significance
*Age (years)*						
Mean/SD	20.5	8.4	21.9	7.2	0.6	Non‐significant
*Sex*						
Male	4	26.7%	9	60%	0.1	Non‐significant
Female	11	73.3%	6	40%		
*Occupation*						
Student	11	73.3%	6	40%	0.06	Non‐significant
Housewife	4	26.7%	2	13.3%		
Driver	0	0%	4	26.7%		
Farmer	0	0%	2	13.3%		
Engineer	0	0%	1	6.7%		

### 3.2 Sex in Different Study Groups

Group I: In the study group, women made up 73.3% and men made up 26.7%.

Group II: Males were 60% and females were 40% of the study group.

There was no statistically significant difference (*p* = 0.1) regarding sex between study groups as demographic characteristics (Table 1).

### 3.3 Occupation in Different Study Groups

Group I: Students were 73.3%, and housewives were 26.7.

Group II: Students were 40%, housewives were 13.3%, drivers were 26.7%, farmers were 13.3%, and engineers were 6.7%.

There were no statistically significant differences regarding occupation between study groups as demographic characteristics (*p* = 0.06) (Table 1).

### 3.4 Vitamin D Level in the Serum

Group I: Vitamin D levels in patients' serum ranged from 4.9 to 18.4 ng/mL with a mean 12.9 and SD 3.8.

Group II: Patients ranged from 20.3 to 64.8 ng/mL with a mean 32.7 and SD 12.8.

Regarding vitamin D levels, there was a statistically significant variation throughout the study groups, with Group II having a higher mean (Table 2).

**TABLE 2:** Comparisons of vitamin D levels in different study groups.

Variables	Vitamin D		*p*	Significance
	Mean	SD		
Group I	12.9	3.8	**< 0.001**	Highly significant
Group II	**32.7**	12.8		

### 3.5 Relation Between Vitamin D Level and Different Occupations Among Study Groups

Farmers had a statistically significantly higher mean level of vitamin D (*p* value < 0.05) (Table 3).

**TABLE 3:** Relation between vitamin D levels and different occupations among study groups.

Occupation	Vitamin D		*p*	Significance
	Mean	SD		
Student	16.9	7.2	**< 0.001**	Highly significant
Housewife	16.8	4.8		
Driver	37.4	7.7		
Farmer	58.5	8.9		
Engineer	27.6	0		

### 3.6 Correlation Between Vitamin D Level With Scar Assessment Before Intervention

Between vitamin D level and scar assessment before intervention (severity of scar), a statistically significant relationship with a *p* value greater than 0.05 did not exist in any case (Table 4).

**TABLE 4:** Correlation between vitamin D levels with scar assessment before intervention.

Variables	Vitamin D level		
	*r*	*p*	Significance
*Before intervention*			
Vascularity	0.06	0.8	Non‐significant
Pigmentation	0.22	0.3	Non‐significant
Pliability	0.02	0.9	Non‐significant
Height	0.24	0.1	Non‐significant
Total score	0.18	0.3	Non‐significant

### 3.7 Comparisons of Scar Assessment Before and After Intervention in 2 Groups

**FIGURE 2:**
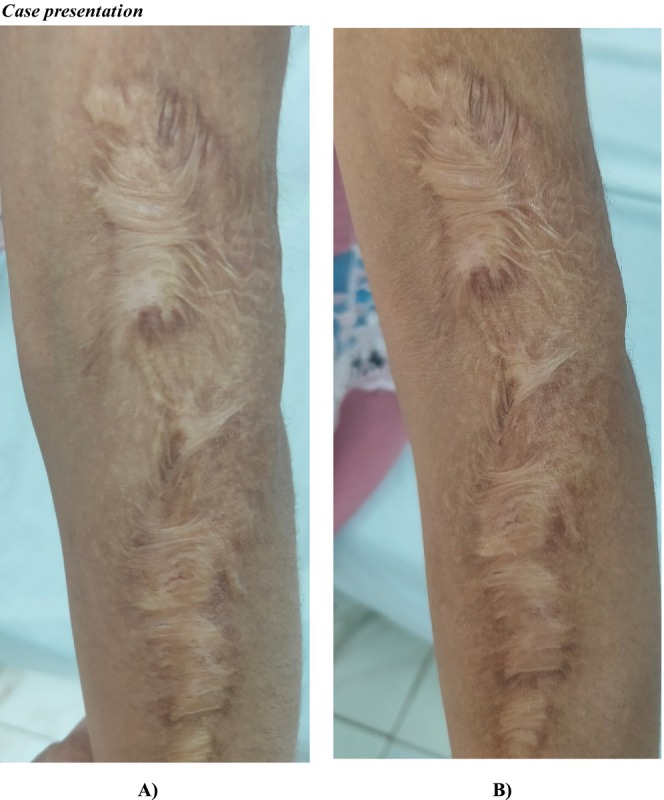
(A) Female group I patient aged 12 with arm keloid. Pre‐treatment VSS:7. (B) Four intralesional and systemic vitamin D3 injections improved VSS to 6 and pliability.

**FIGURE 3:**
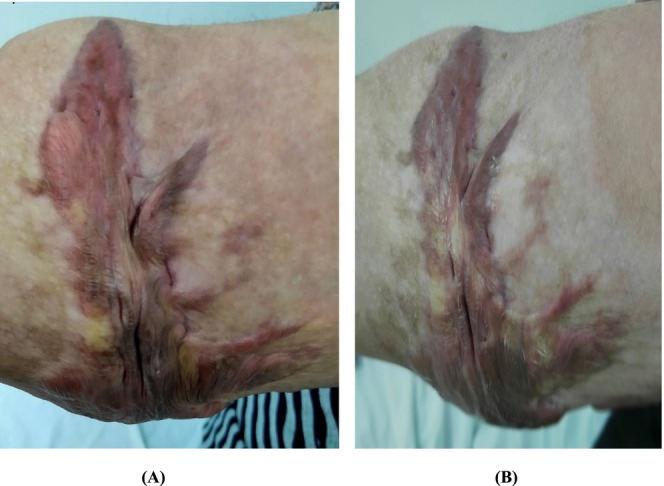
A 30‐year‐old group II woman with an arm keloid. VSS:11 pre‐treatment. (B) Four intralesional vitamin D3 injections improved VSS to 9 for pliability and vascularity only.

Within group comparison, we observed a statistically significant drop in vascularity, pliability, and total score with a *p*‐value < 0.05 after intervention with no change in scare pigmentation and height (Table 5). Between group comparisons, there was no significant difference between all sub‐items of VSS. Figures 2 and 3 showed the morphological changes in two cases in both groups both before and after treatment.

**TABLE 5:** Comparison of scar assessment before and after treatment.

Character	Group 1 (*n* = 15)			Group 2 (*n* = 15)			*p*
	Before treatment	After treatment	*p* ^a^	Before treatment	After treatment	*p* ^a^	After treatment^b^
Vascularity	1.4 ± 1.1	1 ± 0.4	0.03	1.33 ± 0.72	1.13 ± 0.52	0.04	0.7
Pigmentation	1.20 ± 1.01	1.20 ± 1	1	1.67 ± 0.62	1.67 ± 0.62	1	0.1
Pliability	2.8 ± 0.78	1.73 ± 0.59	< 0.001*	2.67 ± 0.62	1.67 ± 0.62	< 0.001*	0.8
Height	1.6 ± 0.51	1.6 ± 0.51	1	1.8 ± 0.41	1.8 ± 0.41	1	0.2
Total score	6.67 ± 2.2	5.5 ± 2.3	< 0.001*	7.47 ± 1.7	6.3 ± 1.5	< 0.001*	0.3

*Note:* Data were presented as mean and standard deviation. (a) Significance within group comparison, (b) significance between group comparison. (*) Significance at *p* < 0.05.

### 3.8 Side Effects of Intralesional Injections of Vitamin D and Follow‐Up

Side effects of the intralesional injection were mild pain, erythema, and swelling at the injection site, which lasted a few hours to 2 days after the injection; but after that, no side effects were observed.

Follow‐up assessment was done after 1 month from the last injection for fear of recurrence, but we found no recurrence rate during that period.

## 4 Discussion

Our research aimed to determine how effective vitamin D injections are (intralesional injection only and both intralesional and systemic injections) on hypertrophic scars and keloids. Also, we evaluate the relation between vitamin D and scar formation severity by measuring serum vitamin D levels using the ELISA technique and VSS before the intervention.

Thirty patients with hypertrophic scars and keloids of both sexes were included, with variable sites, sizes, and thicknesses of keloids and hypertrophic scars among the included cases.

Vitamin D levels were used to categorize patients into two categories.

No statistically significant variations were found between the research groups in terms of sex or age (*p* > 0.05). Vitamin D levels were not significantly different by age or gender (*p* > 0.05). This was consistent with Damanik et al. [7], who found no significant correlation between age and gender in relation to serum 25‐hydroxyvitamin D levels.

On the contrary, Nasri and Ardalan [8], conducted a study on 259 subjects (18–65 years) without any organic problems and found a statistical correlation between age and vitamin D levels.

Research by Kader et al. [9], examined vitamin D levels in serum from 5111 women (75.5% of the total) and 1663 men (24% of the total). The findings demonstrated that elderly people were more likely to have vitamin D deficiency and that women's serum vitamin D levels were much lower than men's.

El Hadidi et al. [6], concluded in a study made by them on 19 patients with keloids of different durations ranging from 1 to 40 years and found that no statistically significant correlation was detected between the assessed serum level of 25‐Hydroxyvitamin D and (age & sex).

In our study, different patients' occupations were included (students, housewives, farmers, drivers, and engineers), and we found a statistically significantly higher mean of vitamin D levels among farmers, with a *p*‐value < 0.001.

This goes ahead with studies made by Sowah et al. [10], and Jeong et al. [11], which found that outdoor workers had higher 25‐hydroxyvitamin D (25‐(O.H.) D) levels than indoor workers.

No statistical correlation was found between serum vitamin D level and severity of scar (total VSS before assessment) with a *p*‐value > 0.05.

Also, El Hadidi et al. [6], found the same results as ours between vitamin D serum level and severity of scar in their study on 19 patients with keloids.

However, Akoh and Orlow [12], and Damanik et al. [7], found that lower serum 25‐hydroxyvitamin D levels increased keloid severity.

According to our study, data revealed a statistically significant decrease in vascularity, pliability, and total VSS with a *p*‐value < 0.05 after the intervention of all cases with no change in scar pigmentation and height. However, there was no statistically significant difference with a *p* value > 0.05 between study groups regarding scar assessment after the intervention.

This is in line with the results of the study by Mamdouh et al. [13], which injected 40 patients with keloid scars 0.2 mL (200 000 IU) of vitamin D per 1 cm lesion once a month into the affected area. Using a high‐resolution ultrasonography in B mode and the VSS, the keloid scars were assessed both before and after therapy. Each patient went through three or four sessions. Intralesional vitamin D injection was found to significantly reduce vascularity, pliability, pigmentation, and keloidal height (*p* value 0.001). In addition, the thickness of ultrasonic keloid scars decreased significantly after therapy (*p* 0.001).

In an in vitro investigation that focused on the mode of action of vitamin D3 on obtained keloid fibroblasts, Ramakrishnan et al. [14], found that after 48 h, keloid fibroblasts treated with vitamin D3 exhibited morphological changes along with a dose‐dependent decrease in cell proliferation in the investigated range of concentrations (5–50 ng/mL). There was a negligible reduction in proliferation after 24 h of therapy. Furthermore, a study conducted by Ramirez et al. [15], revealed that dosage‐dependent regulation of apoptotic‐associated genes by 1,25(O.H.)2D3 suppresses the pro‐fibrotic phenotype of epithelial cells and lung fibroblasts.

According to Ince et al. [16], boosting serum levels of vitamin D to more than 25 ng/mL before scar revision in individuals with H.S. may help reduce scar width by an average of 4 mm. In addition, no scar breadth or score changes were seen in patients who received only vitamin D supplementation. This outcome could be explained by the existence of genetic traits, scar localization, and other factors contributing to the formation of H.S., which the authors could not control in their investigation. Vitamin D injection has a potential role in enhancing the vascularity and pliability of hypertrophic scars and keloids without any effect on pigmentation and height of the scar. Numerous significant regulatory functions, including the regulation of wound repair, cellular differentiation and proliferation, and inflammation, are known to be attributed to vitamin D. These effects are principally mediated by the interaction between vitamin D receptors and active vitamin D, which then initiates gene expression regulation. Vitamin D can cause fibroblast cell death, reduce collagen formation, and delay proliferation of fibrous tissue.

There was no difference in the outcome of intralesional injection of vitamin D in both patients with vitamin D sufficiency and those with deficiency or insufficiency of vitamin D after enhancing their serum vitamin D level by systemic injection of cholecalciferol 200 000 IU injection.

Although there was no statistically significant difference in scar severity before the intervention, systemic administration of vitamin D was justified based on its well‐established role in wound healing, inflammation modulation, and skin regeneration [17, 18]. Previous studies have suggested that vitamin D deficiency may impair normal tissue repair [19, 20], even if baseline severity differences are not observed. Our study aimed to assess whether correcting vitamin D deficiency/insufficiency could influence scar healing outcomes post‐intervention. Additionally, systemic administration ensures adequate bioavailability and addresses any underlying deficiency that might impact healing indirectly.

This outcome suggests that intralesional vitamin D might exert its effects independently of systemic vitamin D status, or that other factors could play a more critical role in scar healing. We recognize that this is an important point and are discussing its implications further in the manuscript.

## 5 Conclusion

Injection of vitamin D on keloids and hypertrophic scars enhances vascularity and pliability in patients with sufficient serum vitamin D levels and those with deficient or insufficient serum vitamin D levels after improving them by systemic injection of vitamin D, without any effect on height and pigmentation of scars.

There is no association between vitamin D deficiency and the tendency for formation or severity of scars.

## 6 Recommendation

1. Vitamin D in hypertrophic scars and keloid treatment requires further assessment and more studies.
2. A combination of other lines of treatment with vitamin D may be required.
3. Multiple sessions of vitamin D injection with a longer follow‐up duration are required to assess their effect on hypertrophic scars and keloids.
4. Further large sample studies should be carried out to determine the role of vitamin D in association with scar formation.

## Author Contributions

All authors made a significant contribution to the work as follows: Conceptualization: A.Y.B., T.A.E.‐M., M.M.B., and N.A. Study design: A.R.H., S.M.E.‐T., A.M.E.‐A., and M.M.I. Data analysis: M.E., M.K., and N.A. Writing original draft: T.A.E.‐M., M.M.B., and A.Y.B. Validation: A.R.H., S.M.E.‐T., A.M.E.‐A., and N.A. Supervision: M.K. and M.E. Writing, review, and editing: A.Y.B., T.A.E.‐M., M.M.B., and M.M.I.

## Ethics Statement

Institutional Review Board of Fayoum University's Faculty of Medicine amended the study protocol and approved it after reviewing it for all ethical and academic issues. The study was approved by the Research Ethics Committee of the Faculty of Medicine, Fayoum University (Approval number: M 415).

## Consent

All patients provided their informed consent.

## Conflicts of Interest

The authors declare no conflicts of interest.

## Data Availability

Upon request, all data can be obtained from the corresponding author.
